# Breakfast Table Talk

**Published:** 1881-03

**Authors:** 


### BREAKFAST TABLE TALK.

Does the wife or mother of every establishment realize how much the happiness of her family for a whole day, not to speak of coming years, is in her keeping; for in her province comes the ordering of her household, and there is no shifting the responsibility of preparing or ordering the breakfast.

Breakfast is the key-note of the day to almost every individual. Those who aver that they care nothing for breakfast, dinner is *their* meal, are the very ones who are unconsciously the victims of an ill-assorted day, from the disastrous effects of an ill-conditioned breakfast.

"Hunger is good sauce," and when stimulated by its demands, the most fastidious may deal peaceably with the distressing agglomeration of food prepared by some housekeepers, miscalled dinner; but from the horrors of a slovenly under or over-done breakfast "saints, angels and ministers of grace defend us."

Too often, especially in the country, does "the madame" rise late, after a day of heavy cares and labors, and perhaps disturbed at night by sick or restless children, and wearily begin her day's work with a spiritless consciousness that it is but another of those that must come while her strength lasts to supplement those already gone before, and she goes about her breakfast with throbbing head and lagging pace. She feels that life is a burden, the hopes of her youth are a failure, and more often her thoughts are straying in gloomy retrospection than occupied in preparing a tidy, wholesome breakfast.

The remains of yesterday's food are indifferently gathered and placed on a smoldering or furious fire, to dry or burn, as circumstances may dictate. A hastily-arranged table is prepared, with the dishes scattered helter-skelter upon it, with half a dozen necessary articles omitted, that some member of the family must rise and procure when needed. Then the family is called to breakfast. They, too, well knowing the style and bill of fare, loiter and delay till the very last grace of patience is exhausted; gather around in sullen silence or with angry altercation, and partake sparingly of such articles as may be necessary to sustain nature. The husband goes to his labor or business illy prepared to meet the demands on his system for want of nutritious food. his feelings disturbed and nervous system awry by reason of fretfulness from some one or more of the family, and he is cross, disagreeable and overbearing in business relations till dinner time.

The children are peevish and unmanageable, often downright quarrelsome for the day—for a broken day is seldom mended, But worse than all, the poor mother takes up *her* burden of employments, with that failing strength and utter giving way of natural spirits that make woeful days for her children, whom she loves better than life, for she is giving her life in the care of them, but for whom the sunshine of neither motherly love nor patience beams that day. And so a whole day is spoiled for a family, and many a life sorrow begun by those who would open their eyes wide in astonishment if told that its genesis was the breakfast table.— *Western Stock Journal.*

